# Exploring How People With Intellectual Disabilities Experience the Therapeutic Alliance: A ‘Best Fit’ Framework Analysis Using Bordin's Model

**DOI:** 10.1111/jar.70187

**Published:** 2026-01-23

**Authors:** Max Whittaker, Andrew Jahoda, Dani Lewis, Dave Dagnan

**Affiliations:** ^1^ Lancashire and South Cumbria NHS Foundation Trust Greenbank Day Centre Preston UK; ^2^ Psychological Medicine, Gartnavel Royal Hospital Glasgow UK; ^3^ Doctorate in Clinical Psychology, the School of Health in Social Science University of Edinburgh Edinburgh UK; ^4^ Cumbria, Northumberland, Tyne and Wear NHS Foundation Trust, Community Learning Disability Services, Unit 9, Lillyhall Business Centre Workington UK; ^5^ University of Cumbria Carlisle UK

**Keywords:** bond, Bordin, goals, intellectual disability, qualitative, tasks, therapeutic alliance

## Abstract

**Background:**

There is an increasing interest in therapeutic alliance within talking therapy for people with intellectual disability. The applicability of frameworks such as Bordin's (1979) model of therapy alliance has not been considered.

**Method:**

A review of qualitative literature on people with intellectual disability's experience of talking therapy identified 23 papers. A ‘best fit’ framework synthesis was used to explore the applicability of Bordin's model to the experiences of people with intellectual disability in therapy.

**Results:**

The analysis supported Bordin's core themes of bond, task and goals. An additional theme was identified concerning the extension of the therapist's role to include active advocacy and support in the lives of people with intellectual disability outside of therapy.

**Conclusion:**

This study identified the experience of people with intellectual disability in therapy as consistent with Bordin's model and suggests that further research specific to the model would be productive.

## 1 Introduction

The evidence base for psychological therapy for people with intellectual disabilities has begun to include both robust trials of specific interventions (e.g., Vereenooghe and Langdon 2013) and consideration of therapy process (e.g., Jahoda et al. 2009). The importance of therapy process has a large evidence base relating to therapy for people without intellectual disabilities, where ‘common factors’ underpinning the successful delivery of therapies have been a particular focus (e.g., Messer and Wampold 2002). Therapeutic alliance is the most frequently studied common factor (Horvath and Symonds 1991).

The key dimensions of the therapeutic alliance continue to be discussed (e.g., Stubbe 2018). However, Bordin's (1979) model is the most widely recognised framework applied to individual (e.g., Ardito and Rabellino 2011) and group (e.g., Pinsof 1988) therapies. Bordin's model is intended to be ‘pan‐theoretical’ and was developed at a time when there was a notable growth in the range of psychotherapeutic approaches being reported (Bordin 1979). Bordin (1979) conceptualised the therapeutic alliance as consisting of a strong, reciprocal bond between patient and therapist, a shared and collaborative understanding of treatment goals, and agreement on the nature of tasks to be undertaken during therapy. A particular strength of this model is that it describes how the personal bond that the therapist develops with the patient supports therapeutic techniques enacted as tasks and goals. Trust is seen as an overarching dimension within the model (Bordin 1979, 254); trust can be conceptualised as the assumption that another person will engage in a ‘joint enterprise competently, in good faith and in a spirit of reciprocity (Arrow 1972)’. Bordin suggests that agreement and ongoing cooperation on goals and tasks between the therapist and patient is most likely to happen if there is a bond grounded in patient and therapist trust in their shared resources (Ardito and Rabellino 2011).

Bordin suggests that the bond influences therapeutic outcomes, not solely because it is healing in and of itself, but also because it enables the patient to accept, adhere to, and believe in the therapeutic techniques and the links made by the therapist between goals and tasks (Prusiński 2022); the specifics of which may vary across therapy approaches (Wampold and Flückiger 2023). Bordin does not describe a hierarchical structure; rather, he acknowledges a multi‐directional interaction between bond, goals, and tasks. Research supports the application of Bordin's model across multiple clinical contexts. For example, Wampold and Flückiger (2023) review evidence that shows that Bordin's model is the most frequently used in the measurement of alliance and that the strength of alliance is consistently shown to be associated with therapy outcome across a broad range of therapy approaches. However, research has rarely considered the applicability of Bordin's model in therapy with people with intellectual disabilities. For example, one small‐scale study (Cameron et al. 2020) examined the use of Bordin's model in talking therapies for people with intellectual disabilities and concluded that therapists and patients with intellectual disabilities experienced the therapeutic alliance as broadly consistent with Bordin's model.

People with intellectual disabilities are likely to be vulnerable to mental health challenges due to the interaction of biological, cognitive, and social factors. For example, people with intellectual disabilities are less likely to experience supportive relationships because of the limitations in opportunities and experiences afforded to people with intellectual disabilities (e.g., Gilmore and Cuskelly 2014). Intellectual disability also remains a stigmatising characteristic (Scior et al. 2013), and the contexts in which individuals with intellectual disabilities live and learn are often characterised by overt social rejection and a lack of personal autonomy and agency (Jahoda et al. 2009). Such factors are clearly relevant to the formation of relationships such as those experienced in therapy, and it is important to explore whether models such as Bordin's are applicable to this patient group.

The absence of specific data on the application of Bordin's model to therapy with people with intellectual disabilities is clear. However, there are several papers that report qualitative data on the general experiences of people with intellectual disabilities in therapy (e.g., Evans and Randle‐Phillips 2020). This paper presents a qualitative meta‐synthesis of such studies, using a framework approach to explore how Bordin's model might be used to understand the nature of the therapeutic relationship for people with intellectual disabilities.

## 2 Materials and Methods

The review protocol was pre‐registered on the International Prospective Register of Systematic Reviews (PROSPERO; protocol number: CRD42023395134) on 03.02.2023. Reporting of this review follows guidelines for systematic reviews (PRISMA‐P; Moher et al. 2015) and qualitative evidence syntheses (ENTREQ; Tong et al. 2012).

### 2.1 Best Fit Framework Analysis

This meta‐synthesis explores the applicability of Bordin's model to qualitative experiences of psychological therapy described by people with intellectual disabilities using a ‘best fit’ framework synthesis (Carroll et al. 2013, 2011). ‘Best fit’ framework analysis (BFFA) uses an already available framework against which data are coded and tested. BFFA takes a critical realist stance (Koopmans and Schiller 2022) and combines an initial deductive framework analysis with subsequent inductive thematic analysis for data that cannot be accommodated within the a priori framework.

BFFA has been used in health research (e.g., Oliver et al. 2008; Carroll et al. 2011, 2013), although it has not thus far been applied widely within clinical psychology and psychological therapy (Jagfeld et al. 2021).

### 2.2 Search Strategy

The current study builds upon search strategies used in a qualitative meta‐synthesis of the experience of people with intellectual disabilities in therapy (Evans and Randle‐Phillips 2020). The search terms used in this study are shown in Table 1. The search was conducted using the CINAHL, PsychINFO, Web of Science and MedLINE databases from 1965 to the present day (searches undertaken on 1st December 2024). The current search strategy identified eight papers in addition to those identified by Evans and Randle‐Phillips (2020).

**TABLE 1:** The search terms used in this study.

Search term	Variation
Intellectual disability	‘Intellectual* disab*’ OR ‘learning disab*’ OR ‘mental* disab*’ OR ‘mentally disabled’ OR ‘cognitive* disab*’ OR ‘mental* retard*’ OR ‘mental* handicap*’ OR ‘mentally handicapped’ OR ‘mental* deficien*’ OR ‘learning difficult*’
Psychological therapy	‘Psycho* therapy’ OR ‘Psycho* treatment’ OR ‘Psycho* intervention’ OR ‘Psychotherap*’ OR ‘psycho‐therap*’ OR ‘therap*’ OR Therapeutic process* OR Psychotherapeutic Process*
Service user experience	Service user experience’ OR ‘Service user views' OR ‘Experiences' OR ‘Views' OR ‘Satisfaction’ OR ‘attitudes' OR ‘Perceptions' OR ‘Patient attitudes' OR ‘Patient Satisfaction’
Qualitative research	‘Qualitative research’ OR ‘Qualitative method*’ OR ‘Thematic analys*’ OR ‘Interpretative Phenomenological Analys*’ OR ‘Grounded Theory*’ OR ‘Discourse Analys*’ OR ‘Content Analys*

### 2.3 Eligibility Criteria

The search identified primary research papers relating to the experiences of people with intellectual disabilities in individual or group‐based psychological therapy. To be included in the synthesis, studies were required to meet the following criteria:

- Published in English
- Published in a peer‐reviewed journal
- Used a qualitative methodology or where studies used a mixed methods approach sufficient detail was provided on the qualitative methodology used and the qualitative research findings to allow analysis
- Involved a psychological talking therapy for emotional or mental health difficulties, defined as ‘group or individual interventions that involve a psychological intervention aimed at the treatment of emotional, behavioural or mental health problems’ (Evans and Randle‐Phillips 2020, 236).
- All participants were aged 18 years or older or the therapy context was clearly an adult service.
- All participants were people with intellectual disabilities confirmed in accordance with the criteria of the services involved in recruitment

### 2.4 Study Selection/Screening Method

**FIGURE 1:**
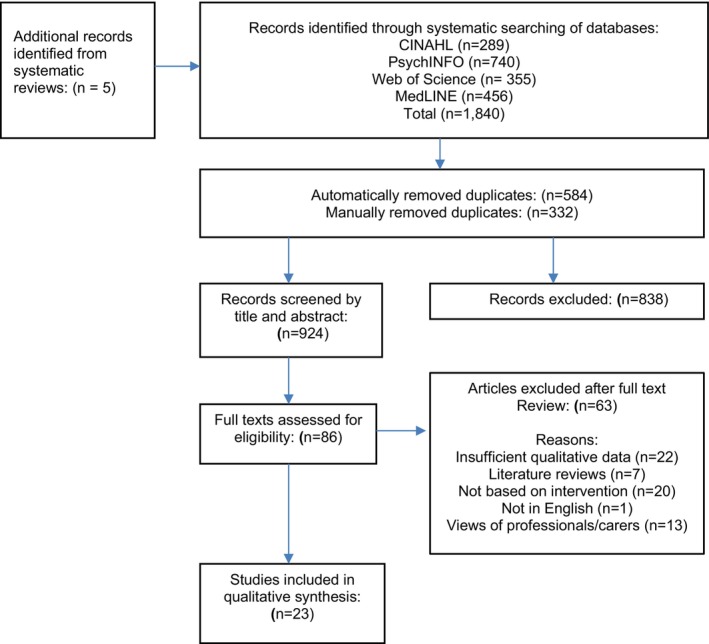
Summary of the search and screening process using a Preferred Reporting Items for Systematic Reviews and Meta‐Analysis (PRISMA) flowchart.

Figure 1 shows a summary of the search and screening process using a Preferred Reporting Items for Systematic Reviews and Meta‐Analysis (PRISMA) flowchart. The searches initially identified 1840 papers, of which 916 were removed as duplicates. The first and last author screened all the unique titles and then abstracts for eligibility. The last author checked 40% of articles discarded at title and abstract stage to ensure that no papers were mistakenly excluded. No discrepant judgements were identified. The first and third author then assessed the full text of eligible studies (*n* = 86), with 23 papers deemed eligible. The last author independently assessed 100% of the papers identified at full text stage with no disagreements identified. Reference and forward citation searches of the included articles found no further papers.

### 2.5 Descriptive Data Extraction

Descriptive data extraction was completed by the first and third authors and core data are presented in Table 2. To ensure accuracy, the extracted data were checked by the last author; no disagreements were identified.

**TABLE 2:** Study characteristics.

Study number	Paper authors and year	Country within which study based	Core aim of study	Participants characteristics (age, sex)	Therapy experienced by participants	Data collection method	Analytic approach
1	Cameron et al. (2020)	United Kingdom (UK)	Exploring the relevance of Bordin's model of adults with intellectual disabilities	3 females, 1 male. Aged 24–48 years.	Mixed: Psychodynamic therapy (2)	Structured	Thematic analysis (Braun and Clarke 2006).
2	Croom et al. (2021)	UK	Exploring the experiences of adults with intellectual disabilities attending a mindfulness‐based group intervention	2 females, 1 male. Aged 19–56 years.	Mindfulness‐based intervention programme	Open‐ended group and individual interviews.	Thematic analysis (Braun and Clarke 2006).
3	Crossland et al. (2017)	UK	Examining the outcomes and experiences of an adapted Dialectic Behaviour	3 females, 1 male.	18‐week DBT skills training group.	Individual semi‐ structured interviews.	Thematic analysis (Braun and Clarke 2006).
			Therapy skills training group for people with intellectual disabilities	Aged 24–48 years.		Mixed methods	
4	Gifford et al. (2012)	UK	What is it like to work with a clinical psychologist of a specialist learning disabilities service?	2 females, 6 males.	Individual psychological therapy (Psychological input varied from 3 to 24 months).	Individual semi‐ structured interviews.	Thematic analysis (Braun and Clarke 2006).
			Views from people with learning disabilities	Aged 27–46 years.			
5	Hardiman et al. (2018)	UK	The experiences of people with intellectual disability in Compassion Focussed Therapy	2 females, 1 male.	Compassion focussed therapy (12–15 week therapy schedule).	Individual semi‐ structured interviews.	Interpretative Phenomenological Analysis (IPA) (Smith et al. 2009).
				Aged 31–48 years.			
6	Hassiotis et al. (2013)	UK	Exploring the experiences of patients with a learning disability with depression and/or anxiety during a manualised CBT programme.	Information not disclosed	Individual CBT for mood disorders (Psychological input occurred over 16 weeks).	Individual semi‐ structured interviews.	Content analysis (Mayring 2023)
7	Hays et al. (2007)	UK	Service user views of group treatment for men with intellectual disability and sexually abusive behaviour	16 males.	Group‐based CBT for individuals with ID with a history of sexual offences. The group occurred over 1 year.	Individual semi‐ structured interviews.	Content Analysis., (Mayring 2023)
				Aged 20–61 years.			
8	Khan and Beail (2013)	UK	Exploring service user satisfaction with individual psychotherapy for people with intellectual disabilities	8 females, 12 males.	Mixed: Psychodynamic therapy (15), integrative counselling (2) and CBT (3).	Individual semi‐ structured interviews.	Thematic analysis (Braun and Clarke 2006).
				Aged 18–64 years.			
9	Lewis et al. (2015)	UK	Exploring how people with Intellectual disabilities experience psychological therapy	5 females, 1 male.	Individual psychological therapy.	Individual semi‐ structured interviews.	IPA (Smith et al. 2009).
				Aged 20–43 years.			
10	Macdonald et al. (2003)	UK	Exploring how people with intellectual disabilities experience group analytic therapy	5 females, 4 males.	Two groups based on psychodynamic group psychotherapy. One group for patients with a history of sexual offending. The other was a women's group. The four male participants had been attending the group for over a year. The five female participants had been in the group for 2–8 months	Individual semi‐ structured interviews.	IPA (Smith et al. 2009)
				Ages of participants not disclosed			
11	MacMahon et al. (2015)	UK	A qualitative study of service users' experiences of a CBT anger management group intervention	3 females, 8 males.	Group‐based CBT for anger management (12 sessions)	Individual semi structured interveiws	IPA (Smith et al. 2009)
				Aged 22–44 years.			
12	Marwood and Hewitt (2012)	UK	Evaluating an anxiety group for people with intellectual disabilities using a mixed methodology	Information not disclosed	Group‐based CBT for anxiety (six sessions)	Individual semi‐ structured interviews	IPA (Smith et al. 2009)
13	Merriman and Beail (2009)	UK	Service user views of long‐term individual psychodynamic psychotherapy	6 males.	Individual psychotherapy (therapeutic input occurred for two or more years).	Individual semi‐ structured interviews.	IPA (Smith et al. 2009)
				Aged 22–45			
14	Pearson et al. (2021)	UK	Exploring the experiences of dialectical behaviour therapy in a community setting for people with intellectual disabilities	8 females, 3 males.	DBT group skills training.	Individual interviews using a flexible interview schedule, suggesting semi‐structured interviews.	IPA (Smith et al. 2009)
				Aged 26–52 years			
15	Pert et al. (2013)	UK	Investigating process issues in CBT from the perspective of people with mild intellectual disabilities	7 females, 8 males.	Individual CBT‐based psychotherapy	Two individual semi‐structured interviews.	IPA (Smith et al. 2009)
				Aged 26–52 years.			
16	Ramsden et al. (2016)	UK	Perceived barriers and facilitators to positive therapeutic change for people with intellectual disabilities	6 males.	Individual psychological therapy completed within the last 3 months.	Individual semi‐ structured interviews.	Thematic analysis (Braun and Clarke 2006).
				Aged 19–43 years.			
17	Roscoe et al. (2015)	UK	Exploring Service users' perspectives of dialectical behaviour therapy in an inpatient unit for women with a intellectual disability	10 females.	DBT‐based skills training programme. Participants had been receiving DBT for varying lengths of time (between 3 and 23 months).	Individual semi‐ structured interviews.	IPA (Smith et al. 2009)
				Aged 19–57 years.			
18	Stenfert‐Kroese et al. (2016)	UK	A pilot study of trauma‐ focussed cognitive‐ behaviour therapy for people with mild intellectual disabilities	3 females, 2 males.	Group‐based trauma‐focused CBT.	Two individual semi‐structured interviews.	IPA (Smith et al. 2009).
				Aged 21–46 years	Therapeutic input occurred over 12 weeks.	Mixed methods.	
19	Thomson and Johnson (2017)	UK	Exploring the experiences of women with intellectual disabilities undergoing dialectical behaviour therapy in a secure service	Information not disclosed.	DBT skills‐based programme.	Two individual semi‐structured interviews.	IPA (Smith et al. 2009).
20	Trustam et al. (2021)	UK	Exploring the recovery experiences of people with intellectual disabilities	3 females, 4 males.	Undisclosed. Participants interviewed following completion of documented mental health treatment.	Individual semi‐structured interviews.	IPA (Smith et al. 2009).
				Aged 20–54 years			
21	Witwer et al. (2025)	USA	Identifying key factors of the therapeutic process from the perspective of people with intellectual disabilities	9 female, 4 male.	Undisclosed. Participants were interviewed following the documented completion of individual mental health treatment	Virtual focus group with semi‐structured interviews	Grounded theory (Corbin and Strauss 2014)
				Aged 19–40 years			
22	Parker et al. (2023)	UK	Exploring how people with intellectual disabilities Experience the therapeutic relationship	6 participants	Individual psychotherapy with a psychologist or counsellor. The main approach used was CBT and psychological input ranged from 6 to 12 months.	Individual semi structured interviews	Interpretative phenomenological analysis (Smith et al. 2009)
				Gender not disclosed			
				Aged 23–46 years			
23	Knight et al. (2019)	UK	Exploring the effectiveness of behavioural activation and guided self‐help interventions for people with intellectual disabilities and depression	17 female, 8 male.	Mixed: Behavioural activation (15) and psycho‐educational intervention (10). Psychological input ranged between 8 and 12 sessions.	Individual semi‐structured interviews	Framework analysis (Ritchie and Lewis 2003)
				Aged 21–66 years			

### 2.6 Study Characteristics

Table 2 outlines the characteristics of the included studies. Sample sizes ranged from three to 25, with a total of 95 males and 88 females, although five studies did not disclose the gender of participants (a total of 41 participants whose gender was unknown). One study in the synthesis was from the United States of America, and all others were from the United Kingdom.

Seven of the studies described experiences of CBT either individually or in a group setting (6, 7, 11, 12, 15, 18, 22), one study exclusively described experience of psychodynamic therapy (10), whilst another study reported 15 people engaging in psychodynamic therapy, two people engaging in integrative counselling and three people engaging in CBT (8). One study described 15 people engaging in behavioural activation and 10 people engaging in guided self‐help (21), four studies described experience of dialectical behaviour therapy (DBT; 3, 14, 17), one study described experience of compassion‐focused therapy (CFT; 5), five studies reported individual ‘psychotherapy’ (4, 9, 13, 16, 23), whilst one study reported experience of ‘therapy with a psychologist’ and did not report specific psychotherapeutic modality (20). Participants in one study (1) had engaged in various therapeutic modalities: psychodynamic therapy (two patients), systemic therapy (one patient), cognitive analytical therapy (CAT; one patient), CBT (one patient) and cognitive behavioural plus acceptance and commitment therapy (ACT; one patient). Seven studies involved group‐based therapy (2, 3, 7, 10, 11, 12, 18), 13 involved individual therapy (1, 4, 5, 6, 8, 9, 13, 15, 16, 20, 21, 22, 23) and three implemented a comprehensive DBT schedule, involving both individual and group‐based therapy (14, 17, 19).

Six studies (1, 6, 10, 11, 13, 16, 23) used formal IQ scores to establish the level of intellectual impairment of participants, indicating that participants within these studies ranged from mild to moderate intellectual disability. Six studies (1, 3, 12, 15, 17, 18) indicated participants had mild intellectual disabilities and five studies reported participants had mild to moderate intellectual disabilities (2, 5, 6, 14, 20), though these did not conduct formal IQ tests. The remaining studies (4, 7, 8, 9, 10, 11, 13, 16, 19, 21, 22) did not comment on the level of intellectual disability of participants but recruited from within intellectual disability services.

Six studies (1, 2, 3, 4, 8, 16) used Thematic Analysis (Braun and Clarke 2006), 13 (5, 9, 10, 11, 12, 13, 14, 15, 17, 18, 19, 20, 22) used Interpretative Phenomenological Analysis (Smith et al. 2009), two studies (6, 7) used Content Analysis (e.g., Mayring 2023), one (21) used Grounded Theory (Birks and Mills 2015), and one (23) used framework analysis (Ritchie and Lewis 2003).

See Table 2 for a complete summary of the characteristics of the studies included in the current synthesis.

### 2.7 Quality Appraisal

Studies were assessed to review methodological rigour but not to exclude studies (Thomas and Harden 2008). The CASP (Critical Appraisal Skills Programme 2018) tool for qualitative papers was used for the included articles. The CASP tool has 10 items, with each item rated as either ‘yes’ (present), ‘no’ (not present) or ‘unclear’ (not clearly reported); a higher number of ‘yes’ ratings suggest a higher level of study quality. The last author rated all papers and the first and third author between them rated all papers. There were 4 disagreements across 230 ratings, indicating a 98.3% agreement, the discrepant ratings were discussed and a consensus on an appropriate rating was reached through discussion. These data are shown in Table 3. Overall, the included studies were of a high quality (all scored > 7), suggesting all used largely appropriate methodologies and reporting. The overall high study quality increases confidence in the current findings.

**TABLE 3:** CASP qualitative study checklist ratings for studies included in the review.

CASP qualitative study quality criteria											
Studies included	Was there a clear statement of the aims of the research?	Is a qualitative methodology appropriate?	Was the research design appropriate to address the aims of the research?	Was the recruitment strategy appropriate to the aims of the research?	Was the data collected in a way that addressed the research issue?	Has the relationship between researcher and participants been adequately considered?	Have ethical issues been taken into consideration?	Was the data analysis sufficiently rigorous?	Is there a clear statement of findings?	Is the research valuable?	Total score
Cameron et al. (2020)	1	1	1	1	1	1	1	1	1	1	10
Croom et al. (2021)	1	1	1	1	1	0	1	1	1	1	9
Crossland et al. (2017)	1	1	1	1	1	0	0	1	1	1	8
Gifford et al. (2012)	1	1	1	1	1	1	1	1	1	1	10
Hardiman et al. (2018)	1	1	1	1	1	0	1	1	1	1	9
Hassiotis et al. (2013)	1	1	1	1	1	0	1	1	1	1	9
Hays et al. (2007)	1	1	1	1	1	1	1	0	1	1	9
Khan and Beail (2013)	1	1	1	1	1	0	0	1	1	1	8
Lewis et al. (2015)	1	1	1	1	1	1	1	1	1	1	10
Macdonald et al. (2003)	1	1	1	1	1	0	1	1	1	1	9
MacMahon et al. (2015)	1	1	1	1	1	1	1	1	1	1	10
Marwood and Hewitt (2012)	1	1	1	1	1	0	1	1	1	1	9
Merriman and Beail (2009)	1	1	1	1	1	1	1	1	1	1	10
Pearson et al. (2021)	1	1	1	1	1	1	1	1	1	1	10
Pert et al. (2013)	1	1	1	1	1	0	1	1	1	1	9
Ramsden et al. (2016)	1	1	1	1	1	1	1	1	1	1	10
Roscoe et al. (2015)	1	1	1	1	1	1	1	1	1	1	10
Stenfert‐Kroese et al. (2016)	1	1	1	1	1	0	1	1	1	1	9
Thomson and Johnson (2017)	1	1	1	1	1	1	1	1	1	1	10
Trustam et al. (2021)	1	1	1	1	1	0	1	1	1	1	9
Witwer et al. (2025)	1	1	1	1	1	0	0	1	1	1	8
Parker et al. (2023)	1	1	1	1	1	1	1	0	1	1	9
Knight et al. (2019)	1	1	1	1	1	0	1	0	1	1	8

### 2.8 Data Synthesis

The current study examined whether the therapeutic alliance as described in Bordin's model fits the experience of people with intellectual disabilities. BFFA uses an a priori framework as a scaffold around which novel findings may be brought together and organised (Carroll et al. 2013). Analysis was at the level of the statement in results sections, statements could be phrases, sentences or paragraphs that presented a concept relevant to the therapeutic alliance. A deductive framework analysis was initially applied to the qualitative data to determine the extent to which the data could be integrated into the categories of bond, goals and tasks. Where data from the studies were judged to related to alliance but could not be placed into Bordin's themes or where there were sufficient data within a theme, a second order inductive reflexive thematic analysis (Braun and Clarke 2019) was carried out.

The presence of a theme suggests codes have several occurrences across the data (see Table 4), although determining the presence of a theme ultimately requires the judgement of the researchers. Such judgements were based on an iterative and discursive process involving discussion between the researchers, to determine their reasonableness and robustness.

## 3 Results

### 3.1 Synthesis

Table 4 summarises the best fit framework synthesis. The table identifies themes identified with the core elements of Bordin's model of alliance and the papers which contributed to the themes. Three primary themes were identified within the category of bond, one of which has two subordinate themes.

**TABLE 4:** Summary of best fit framework synthesis.

Superordinate themes of Bordin's model	Primary themes	Subordinate themes	Studies informing constructs
Bond	Trust		1, 14, 15, 17, 18, 20
	A distinctive relationship		1, 2, 4, 5, 9, 15, 18, 20
	Therapy is safe	Confidentiality and containment	3, 4, 5, 6, 7, 9, 10, 15, 17, 20
		No threat of negative evaluation	1, 4, 7, 10, 12, 13, 14, 15, 20
Therapy tasks	Specific therapeutic tasks		3, 4, 5, 6, 7, 9, 11, 12, 14, 1719, 20
	The task of collaboration		1, 9, 10, 13, 14, 15
Therapy goals	Explicit goals		1, 4, 9, 15, 17
	Emerging goals		1, 4, 9, 12, 13, 14, 17, 18, 20
The alliance in context: extending the relationship			1, 4, 9, 13, 15, 16, 17, 22, 23

#### 3.1.1 Superordinate Theme: Bond

##### 3.1.1.1 Primary Theme: Trust

Trust is seen as a central feature of the bond:

I just got a relationship with people who I trust and talk to, it takes for me to trust somebody quite a lot. I have been here now a long time, and [therapist] has been there since day one. (Roscoe et al. 2015, 272)

Trust was particularly important to patients with previous experiences of unfair treatment and an absence of other interpersonal relationships based on trust (Trustam et al. 2021). As one patient stated:

I can never ever trust people. That's what I say to them. You can never trust. This person could be your favourite friend, right. And you don't know what that friend's going to say to the next person. See that's why I have so many secrets, I can't tell nobody. Because I don't know who they're going to tell on to. (Macdonald et al. 2003, 443)

##### 3.1.1.2 Primary Theme: A Distinctive Relationship

The relationship with the therapist was identified as distinct from relationships with family, friends and other professionals outside of therapy:

He's like a counsellor but he's like a friend. You could talk to him about anything and he just listens and helps you out […] he's been like a guardian angel. (Cameron et al. 2020, 173)

Another patient contrasted the relationship with their therapist with that of family members, in a way that underscored their sense of connectedness:

It's somebody that understands how you feel. Because for all my family talk to me, but they don't talk to me in the way I want them to talk to me. (Pert et al. 2013, 362)

##### 3.1.1.3 Primary Theme: Therapy Is Safe

There was a strong theme of the alliance as a ‘safe’ experience, this was particularly in contrast to the world outside of therapy. This was seen in two sub‐ordinate themes.

###### 3.1.1.3.1 Subordinate Theme: Confidentiality & Containment

The private and containing quality of the therapeutic alliance was important for many patients. Patients understood that disclosures within the therapeutic encounter would not be shared with others:

It's better to talk to people one to one, then you can talk to him about how you're feeling, what your problems are and how you can sort them out without having to talk about them in front of your parents. (Trustam et al. 2021, 256)

Patients' confidence in confidentiality appeared to generate the trust that is required for genuine relational depth to emerge (Gifford et al. 2012; Lewis et al. 2015). The value attached to confidentiality was particularly evident in group settings and in patient cohorts with forensic histories (Hays et al. 2007). Some patients expressed their preference for one‐to‐one sessions (Thomson and Johnson 2017), due to their increased confidence in the private quality of one‐to‐one conversations:

I felt a bit more comfortable one to one, because you can talk about things that are private and confidential. Any, like…eh problems that you've got that you don't want anybody else to know because it's private, you know. (Pert et al. 2013, 363)

###### 3.1.1.3.2 Subordinate Theme: No Threat of Negative Evaluation

Patients valued the safety of not being met with negative responses. One patient, for example, described being uncertain of the response they would elicit from their therapist:

I was a bit nervous. I didn't know what to say. I got a bit tight, scared of what he might do to me…. (Merriman and Beail 2009, 44).

Some patients had learned to anticipate negative evaluation and hostility due to experiences of victimisation at home or in the community (Trustam et al. 2021) and general concerns about being met with mockery or being laughed at (Macdonald et al. 2003), and thus, placed emphasis on the importance of a non‐judgmental ethos (Marwood and Hewitt 2012):

It's none of that ‘you better watch what you're saying’. With (therapist) you can just let it all hang (out). (Pert et al. 2013, 363)

#### 3.1.2 Superordinate Theme: Therapy Tasks

##### 3.1.2.1 Primary Theme: Specific Therapeutic Tasks

Tasks were often associated with specific therapeutic approaches (e.g., MacMahon et al. 2015):

My tasks were, if I was worried about something, go through it myself, self‐help, then it was like read things on the wall, then he suggested writing things down and passing it onto support workers. (Knight et al. 2019)

Patients were able to link tasks to their difficulties and goals, such as establishing routines, exposure to stimuli associated with specific phobias or emotional processing and located these in the trust they have in the therapist (Hassiotis et al. 2013). This specific link between task and goals and the observation that this was recognised by patients is of particular importance in Bordin's model (Bordin 1979). As one patient commented:

…like everyday she has me do this circle (which) has these different emotions and she has me colour on the emotions. Like pick a colour each emotion is…but the whole point of doing that daily thing is she's trying to help me form a routine because I struggle a lot with keeping routines. (Witwer et al. (2025))

##### 3.1.2.2 Primary Theme: The Task of Collaboration

Patients across studies placed emphasis on collaboration within the relationship with their therapists (Cameron et al. 2020):

[the therapist] would play it by ear, depending on what I wanted to talk about and what I didn't want to talk about. (Pearson et al. 2021, 290)

For other patients, collaboration was implicit in their descriptions, of actively participating, rather than adopting a passive relational style:

I got up and done all the drawings on the whiteboard (…) And the writing on the whiteboard (…) I asked if I could get up and do some writing. (Pearson et al. 2021, 287)

Patients emphasised their need to assume personal responsibility to improve their lives by actively collaborating with the content of psychological therapy:

Well, it's not really what she (the psychologist) can change, it's mainly what I can change, I mean she can give me all the advice in the world cause virtually that's what she was doing but it was up to me to take on board what she was saying and do something about it myself, so it was all about me. (Gifford et al. 2012, 118)

#### 3.1.3 Superordinate Theme: Therapy Goals

##### 3.1.3.1 Primary Theme: Explicit Goals

Explicit goals were collaboratively identified by patients and therapists.

SC: So how did you decide what steps you would do first?

CLIENT: (Daughter) is the step first thing, then we said we were going to start doing the confidence build up and work up to the top. (Cameron et al. 2020, 174)

For some patients, goals were sometimes phrased in a broader, non‐specific terms, with one patient expressing specific goals in the following terms:

Um, making me feel happier at home to make me think more for myself. (Gifford et al. 2012, 118)

Other patients identified goals such as a desire for positive changes in relationships and to experience more social opportunities. For example:

Change the way others see me; To do things for myself; Feel better about myself; To get a job; Build my confidence; To get something to do; Stop letting things get to me; To get out more; and Stop being aggressive; meet new people; Express myself better; To make friends. (Pert et al. 2013, 364)

##### 3.1.3.2 Primary Theme: Emerging Goals

Patients described a range of goals and achievements that seemed to have emerged during therapy and, at times, and an emerging frustration with how hard it was to make changes. For example, patients reported experiencing more gratifying social interactions following therapy, and emphasised improvements in their interpersonal relationships. A greater feeling of acceptance was seen as an important aspect of recovery (Stenfert‐Kroese et al. 2016). One patient commented:

I think the difference is I can talk more, I can talk to people a bit, a bit better—like my friends …, which is now. I think, back in the past it was more difficult for me to talk to anyone. (Trustam et al. 2021, 257)

Patients often stated these as outcomes that were compared to their pre‐therapy experience:

I'm not more stressed than I used to be. I feel a lot more confident, well actually I wouldn't say I'm more confident in myself, but more like happier in myself. (Cameron et al. 2020, 174)

However, some patients identified a degree of fragility in the achievements identified and were cautious about the maintenance of changes:

I just take one step at a time and see how it goes I can't really think that far ahead. (Pert et al. 2013, 365)

Other patients identified that goals were hard to identify and achieve

I don't know because I've been seeing (therapist) for a year now. So I don't think I will get any better. (Cameron et al. 2020, 174)

#### 3.1.4 Superordinate Theme: The Alliance in Context: Extending the Relationship

Patients described their therapist as acting in a bridging and coordinating capacity to ensure they received appropriate care from within their wider support system. This was considered a central aspect of how therapy worked by many patients and reflected the trust that was established in the therapist:

If I had a problem with anything I would just ring her and she would get back to me and help me get in touch with the right people to sort it. (Ramsden et al. 2016, 253)

Patients identified how their therapist would act in a coordinating capacity, attending meetings with others within their support system, including staff and family members. One patient described how one of the tasks of therapy was to identify a strategy to help ensure safety and manage risk‐taking behaviours:

I text staff and asked them to call me and they will call me and we will have a certain time to go out and they will call me within that hour to see that I'm safe. (Gifford et al. 2012, 118)

Patients experienced such systemic involvement as facilitating therapy and it emphasised the trust they had in the therapist to act on their behalf. However, some patients described a more passive relational style, in which the therapist solved their problems, rather than helping the patient develop problem‐solving skills:

X sorts my problems out. I've had problems the last three weeks and he wrote them down and sorted it out. I leave it to him to sort things out. (Merriman and Beail 2009, 44)

Extending the therapy alliance into the lives of people with intellectual disabilities outside of the therapy session was often enacted by members of the patients support network being present in therapy. Having someone they knew in sessions often helped to overcome their anxieties about therapeutic sessions and facilitated and scaffolded the therapeutic alliance:

It helped having a supporter there because I was stuck. My auntie come up with some ideas. It was good, when I was stuck she came out with ideas. (Knight et al. 2019)

However, for some patients, the presence of a supporter was counterproductive and compromised the safety that was a key part of the alliance, highlighting the additional complexity within therapeutic process where others are present:

I'm just a bit wary about (support worker), I don't want her to listen. (Parker et al. 2023)

## 4 Discussion

This qualitative synthesis examined whether Bordin's model (1979) was suitable as a ‘best fit’ framework when describing the dimensions of alliance with a therapist for people with intellectual disabilities. Consistent with Cameron et al. (2020), the current study suggests that the experience of alliance can be constructed within this framework. The bond was particularly central to the experience of patients with intellectual disabilities and was formulated as having three components: *trust*, *distinctiveness*, and *safety*. *Safety* also comprised two subthemes: *confidentiality and containment*, and *no threat of negative evaluation*. Tasks associated with the activity of *specific therapies* and associated with specific goals were also identified as were more general emerging tasks of *collaboration*. Goals were discussed both as *specific, and related to tasks*, and as *emergent throughout therapy*. Finally, an additional theme of the therapy alliance enacted within the wider context of the person's life was identified. This theme suggests Bordin's model may need to be expanded to account for the social and interpersonal context of intellectual disabilities, with some patients describing the alliance as having an active role in enabling therapy to influence their wider life experiences. Whilst a specific theme of the therapy being active in the wider context of people's lives enabled by the therapeutic relationship emerged in this study many of the aspects of Bordin alliance domains found to be inportant in this study also reflect the context of the lives of people with intellectual disability. For example, personal safety is widely recognised as central to psychotherapy (Podolan and Gelo 2023), but an emphasis on safety is particularly salient to the lives of people with intellectual disabilities, as they are more likely to experience rejection, stigmatisation and victimisation (Dagnan and Waring 2004).

The social and cultural context of the lives of people with intellectual disabilities is important in understanding the nature and focus of the therapeutic relationship and has been identified as an important additional dimension to Bordin's model for other populations (e.g., Ross et al. 2008; Vasquez 2007). Specifically, when working with people with intellectual disabilities a focus on autonomy and personal responsibility needs to be appropriately balanced with a recognition that people with intellectual disabilities often do not have high levels of agency and autonomy and need support to enact changes in their lives. We have seen these activities as a distinctive element of therapy but they could also be seen as an overarching factor across the bond, goal, and task framework. If the therapist is taking on a broader support for the patient with an intellectual disability as part of the therapeutic activity this is inevitably part of their relationship with the patient. In addition to the therapist's involvement outside of the therapy session there are also many examples of the patient's supporters or significant others playing a part in the tasks and goals of therapy (e.g., Knight et al. 2019). There are several potential benefits of this approach (e.g., Surley et al. 2024), however, the demands on the therapist and supporters in adopting therapeutic roles in wider aspects of the patient's life need careful consideration and has implications for factors such as confidentiality and therapist training (e.g., Surley et al. 2024). This needs further research.

There is overlap between the themes relating to Bordin's core categories. Bordin specifically acknowledged the substantive overlap between tasks, goals and interpersonal processes, noting that tasks do not just involve the specific activities of the therapy approach but also as including ‘*empathic understanding, communicating, interpreting [and] self‐disclosing’ (1979, 254)*. The importance of collaborative task and goal setting is emphasised in several studies and has a significant impact upon outcome (Wampold 2015). In this study goals were described as both associated with specific therapy tasks and as emergent and, at times, expressed as outcomes of therapeutic activity. This is consistent with wider studies of goals in therapy; for example, Oddli et al. (2021, 1320) report that therapy patients often do not identify clear initial goals but that therapists ‘*align with patient directionality in a forward driven, gradually evolving process’*. It is possible that goals and tasks for people with intellectual disabilities in therapy are more likely to be emergent as taking time to socialise the patient into therapy models and relationships is likely to be particularly important for this patient group (Dagnan et al. 2013).

Previous studies found that patients and therapists may have different perspectives on the therapeutic alliance (e.g., Atzil‐Slonim et al. 2015) and there has been surprisingly little research on how therapists working with people with intellectual disabilities experience the therapy alliance. Cameron et al. (2020) demonstrated that therapists' reports of therapy were consistent with the three elements of Bordin's (1979) model, although the study was based only on six therapists. Oudshoorn et al. (2023) report the factor structure of the Working Alliance Inventory (Horvath and Symonds 1991), which is a measure of therapy alliance that is based upon the three elements of Bordin's (1979) model. They used confirmatory factor analysis on 199 responses from therapists and staff supporting people with intellectual disabilities and found that the expected three components were present in their data. The limited research suggests that Bordin's model fits the perspective of therapists and staff working with people with intellectual disabilities, however, research exploring the different emphasis placed on the model dimensions between therapists and patients is important.

This review has clear limitations. The aims of the original studies were typically not specifically related to alliance. Care was taken in this synthesis to use statements and ideas that had a clear relation to the alliance. However, had researchers in the initial studies framed their enquiries within Bordin's tripartite model, patients may have emphasised aspects of their experience that corresponded more or less closely to Bordin's model. Further, the current synthesis integrated the experiences of patients receiving both individual and group psychotherapy. There are good reasons to think that including both individual and group‐based psychotherapies is worthwhile as Bordin's model has previously been used to describe individual, couples and group therapies (Pinsof 1988; Pinsof and Catherall 1986). However, there may be differences in emphasis in how elements of the model present in different therapy types. This study was concerned with talking therapy with adults with intellectual disabilities. Therapy alliance in therapy such as with children with intellectual disabilities and in creative therapies should also be explored.

The current study identified broad areas of patient experience that are consistent with Bordin's model. It was notable that it was possible to extract more detailed data relating to the bond than relating to goals and tasks. The superordinate themes of trust and safety, emphasise the experience of therapy as a different type of relationship to those typically experienced for people with intellectual disabilities. Tasks and goals were a salient aspect of the experience of alliance and were described as both explicit formal collaborative processes and as emergent properties of the therapeutic process. Bordin's model may not entirely accommodate the social context of intellectual disability, and this may require therapists to appropriately balance goals relating to independence and autonomy against the need to assume a proactive approach to advocacy and problem solving on behalf of people with intellectual disability.

## Funding

The authors have nothing to report.

## Ethics Statement

This is a systematic review; data are available in published papers.

## Consent

The authors have nothing to report.

## Conflicts of Interest

The authors declare no conflicts of interest.

## Data Availability

The data that support the findings of this study are available in already published papers.
